# {μ-2-[4-(Benzothia­zol-2-yl)benz­yl]-2-aza­propane-1,3-dithiol­ato-1:2κ^4^ *S*,*S*′:*S*,*S*′}bis­[tricarbonyl­iron(I)]

**DOI:** 10.1107/S1600536812006861

**Published:** 2012-02-24

**Authors:** Shang Gao, Qian Duan, Da-yong Jiang

**Affiliations:** a School of Materials Science and Engineering, Changchun University of Science and Technology, No. 7989, Weixing Road, Changchun 130022, People’s Republic of China

## Abstract

The title compound, [Fe_2_(C_16_H_14_N_2_S_3_)(CO)_6_], was prepared as the biomimetic model for the active site of iron-only hydrogenase. The structure is similar to the diiron subsite of the iron-only hydrogenase active site, and contains a diiron-aza­dithiol­ate moiety in which a boat six-membered ring is fused with a chair six-membered ring. The substituted benzyl group attached to the bridging N atom resides in an equatorial position. The sum of the C—N—C angles around this N atom [331.9 (12)°] indicates *sp* ^3^ hybridization.

## Related literature

For general background, see: Cammack (1999 ▶); Evans & Pickett (2003 ▶); Peters *et al.* (1998 ▶); Nicolet *et al.* (1999 ▶). For the crystal structure of the natural enzyme, see: Nicolet *et al.* (2000 ▶); Frey (2002 ▶). For enzyme synthetic models, see: Felton *et al.* (2009 ▶); Tard & Pickett (2009 ▶).
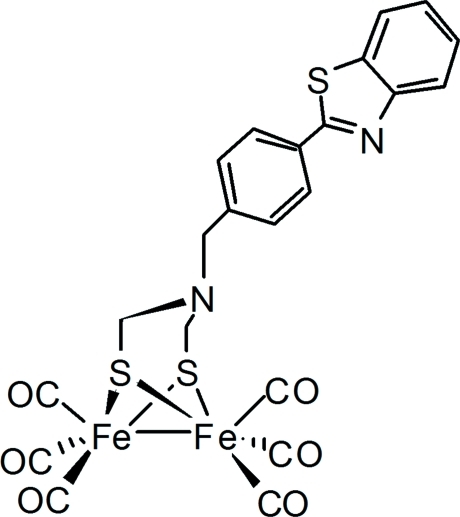


## Experimental

### 

#### Crystal data

[Fe_2_(C_16_H_14_N_2_S_3_)(CO)_6_]
*M* *_r_* = 610.23
Orthorhombic, 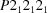

*a* = 6.651 (3) Å
*b* = 14.208 (7) Å
*c* = 25.854 (12) Å
*V* = 2443.2 (19) Å^3^
*Z* = 4
Mo *K*α radiation
μ = 1.49 mm^−1^
*T* = 273 K
0.25 × 0.08 × 0.07 mm

#### Data collection

Bruker SMART CCD area-detector diffractometer
Absorption correction: multi-scan (*SADABS*; Bruker, 1997 ▶) *T*_min_ = 0.332, *T*_max_ = 0.905
11654 measured reflections
3991 independent reflections
3434 reflections with *I* > 2σ(*I*)
*R*_int_ = 0.030

#### Refinement

*R*[*F* ^2^ > 2σ(*F* ^2^)] = 0.027
*wR*(*F* ^2^) = 0.052
*S* = 0.98
3991 reflections
316 parameters
H-atom parameters constrained
Δρ_max_ = 0.22 e Å^−3^
Δρ_min_ = −0.16 e Å^−3^

### 

Data collection: *SMART* (Bruker, 1997 ▶); cell refinement: *SAINT-Plus* (Bruker, 2001 ▶); data reduction: *SAINT-Plus*; program(s) used to solve structure: *SHELXS97* (Sheldrick, 2008 ▶); program(s) used to refine structure: *SHELXL97* (Sheldrick, 2008 ▶); molecular graphics: *SHELXTL* (Sheldrick, 2008 ▶); software used to prepare material for publication: *SHELXTL*.

## Supplementary Material

Crystal structure: contains datablock(s) global, I. DOI: 10.1107/S1600536812006861/lr2050sup1.cif

Structure factors: contains datablock(s) I. DOI: 10.1107/S1600536812006861/lr2050Isup2.hkl

Additional supplementary materials:  crystallographic information; 3D view; checkCIF report

## Figures and Tables

**Table:** 

Fe1—S1	2.2485 (11)
Fe1—S2	2.2487 (11)
Fe1—Fe2	2.5013 (12)
Fe2—S1	2.2465 (11)
Fe2—S2	2.2534 (11)
N1—C8	1.445 (3)
N1—C7	1.448 (4)
N1—C9	1.472 (3)

**Table:** 

C1—Fe1—Fe2	147.16 (10)
C6—Fe2—Fe1	148.57 (11)
C8—N1—C7	111.8 (2)
C8—N1—C9	110.6 (2)
C7—N1—C9	109.4 (2)

**Fig. 1.**
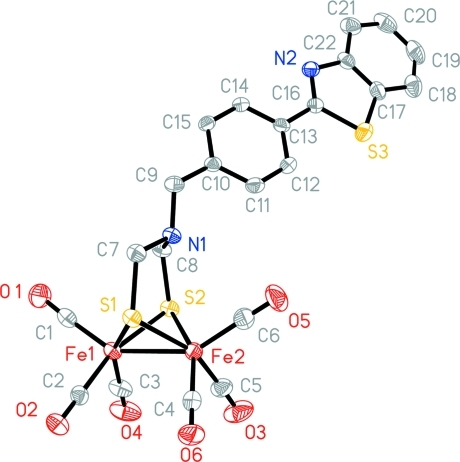
The molecular structure of the title compound, with displacement ellipsoids drawn at 30% probability level.

## supplementary crystallographic information

### Comment

The iron-only hydrogenases are important enzymes which catalyze the reduction of protons to molecular hydrogen in microorganisms (Cammack, 1999, Evans & Pickett, 2003). The crystal structure elucidation indicates that the active site of iron-only hydrogenase contains carbon monoxide ligands and an azadithiolate bridging two iron centers (Nicolet *et al.*, 2000, Frey, 2002). Small synthetic model compounds have turned out to be an alluring topic for the purpose to understand the mechanisms of the enzymes (Felton *et al.*, 2009, Tard & Pickett, 2009). The title compound was prepared to mimic structurally the active site of iron-only hydrogenases. Herein we report its crystal structure.

The structure of title compound is similar to the active site of iron-only hydrogenases, with a butterfly architectonic Fe_2_S_2_ core and the usual distorted square-pyramidal geometry around the iron centre. The length of Fe—Fe bond [2.5013 (12) Å] is somewhat shorter than those in the structures of natural enzymes (*ca* 2.6 Å) (Peters *et al.*, 1998, Nicolet *et al.*, 1999). The N-substituted azadithiolate ligand is *η*^2^:*η*^2^-coordinated to the Fe(CO)_3_ moieties to form two fused six-member rings. Ring Fe1—S1—C7—N1—C8—S2 has a chair conformation, while ring Fe2—S1—C7—N1—C8—S2 has a boat conformation. The substituted benzyl ring attached to N1 atom resides in an equatorial position and the nitrogen lone electron pair is in an axial position. As a result, the C1—Fe1—Fe2 angle [147.16 (11)°] and the C6—Fe2—Fe1 angle [148.57 (11)°] are almost equal. The sum of C—N—C angles around N1 atom is 331.9 (12)°, roughly consistent with an *sp*^3^-hybridization of N1 atom.

Selected bond distances and angles are summarized in Table 1, and the molecular structure of the title compound is shown in Fig.1.

### Experimental

All reactions and operations related to the title compound were carried out under a dry, prepurified nitrogen atmosphere with standard Schlenk techniques. All solvents were dried and distilled prior to use according to standard methods. *N,N'*-bis(hydroxymethyl)-(4-benzothiazole)-benzylamine (2.25 g, 7.5 mmol), prepared from 4-benzothiazole-benzylamine and HCHO-H_2_O, was added to a degassed THF solution (30 ml) of (µ-HS)_2_Fe_2_(CO)_6_, freshly derived from (µ-S_2_)Fe_2_(CO)_6_ (1.38 g, 4 mmol), reacted with LiEt_3_BH (1 *M* solution in THF, 8 ml, 8 mmol) and F_3_CCO_2_H (0.6 ml, 8 mmol) at 195 K. The reaction mixture was stirred for 1 h at 195 K, and allowed to warm up to room temperature. The solvent was removed *in vacuo* and the resulting red solid was purified by column chromatography (silica, 20% dichlorometnane in hexane as eluent). The title compound was obtained in 72% yield (1.77 g). Recrystallization in the CH_2_Cl_2_/hexane solution afforded crystals suitable for X-ray study.

### Refinement

The H atoms attached to C were placed in geometrically calculated positions (C—H = 0.93–0.97 Å) and refined as riding, with *U*_iso_(H) = 1.2*U*_eq_(C).

### Crystal data

**Table:**

[Fe_2_(C_16_H_14_N_2_S_3_)(CO)_6_]	*F*(000) = 1232
*M**_r_* = 610.23	*D*_x_ = 1.659 Mg m^−^^3^
Orthorhombic, *P*2_1_2_1_2_1_	Mo *K*α radiation, λ = 0.71073 Å
Hall symbol: P 2ac 2ab	Cell parameters from 8061 reflections
*a* = 6.651 (3) Å	θ = 2.8–23.5°
*b* = 14.208 (7) Å	µ = 1.49 mm^−^^1^
*c* = 25.854 (12) Å	*T* = 273 K
*V* = 2443.2 (19) Å^3^	Needle, red
*Z* = 4	0.25 × 0.08 × 0.07 mm

### Data collection

**Table:**

Bruker SMART CCD area-detector diffractometer	3991 independent reflections
Radiation source: fine-focus sealed tube	3434 reflections with *I* > 2σ(*I*)
Graphite monochromator	*R*_int_ = 0.030
phi and ω scans	θ_max_ = 24.5°, θ_min_ = 2.1°
Absorption correction: multi-scan (*SADABS*; Bruker, 1997)	*h* = −7→7
*T*_min_ = 0.332, *T*_max_ = 0.905	*k* = −15→16
11654 measured reflections	*l* = −22→30

### Refinement

**Table:**

Refinement on *F*^2^	0 restraints
Least-squares matrix: full	H-atom parameters constrained
*R*[*F*^2^ > 2σ(*F*^2^)] = 0.027	*w* = 1/[σ^2^(*F*_o_^2^) + (0.0229*P*)^2^] where *P* = (*F*_o_^2^ + 2*F*_c_^2^)/3
*wR*(*F*^2^) = 0.052	(Δ/σ)_max_ = 0.002
*S* = 0.98	Δρ_max_ = 0.22 e Å^−^^3^
3991 reflections	Δρ_min_ = −0.16 e Å^−^^3^
316 parameters	

### Special details

**Table:**

Geometry. All e.s.d.'s (except the e.s.d. in the dihedral angle between two l.s. planes) are estimated using the full covariance matrix. The cell e.s.d.'s are taken into account individually in the estimation of e.s.d.'s in distances, angles and torsion angles; correlations between e.s.d.'s in cell parameters are only used when they are defined by crystal symmetry. An approximate (isotropic) treatment of cell e.s.d.'s is used for estimating e.s.d.'s involving l.s. planes.
Refinement. Refinement of *F*^2^ against ALL reflections. The weighted *R*-factor *wR* and goodness of fit *S* are based on *F*^2^, conventional *R*-factors *R* are based on *F*, with *F* set to zero for negative *F*^2^. The threshold expression of *F*^2^ > σ(*F*^2^) is used only for calculating *R*-factors(gt) *etc*. and is not relevant to the choice of reflections for refinement. *R*-factors based on *F*^2^ are statistically about twice as large as those based on *F*, and *R*- factors based on ALL data will be even larger.

### Fractional atomic coordinates and isotropic or equivalent isotropic displacement parameters (Å^2^)

**Table:**

	*x*	*y*	*z*	*U*_iso_*/*U*_eq_	
Fe1	0.16578 (7)	0.28420 (3)	0.813925 (15)	0.04281 (12)	
Fe2	0.16580 (7)	0.44576 (3)	0.775496 (16)	0.04460 (12)	
S1	−0.00452 (12)	0.32359 (6)	0.74198 (3)	0.04209 (19)	
S2	0.43111 (11)	0.34726 (5)	0.77264 (3)	0.0433 (2)	
S3	1.14717 (17)	0.58001 (6)	0.54946 (3)	0.0657 (3)	
N2	1.2033 (4)	0.42166 (17)	0.50317 (9)	0.0503 (7)	
C22	1.3625 (5)	0.4801 (2)	0.48913 (12)	0.0512 (8)	
N1	0.3099 (4)	0.32042 (16)	0.67095 (8)	0.0379 (6)	
O2	−0.2259 (4)	0.26309 (18)	0.86511 (9)	0.0650 (7)	
C15	0.6908 (5)	0.2851 (2)	0.56797 (10)	0.0424 (7)	
H15A	0.6649	0.2220	0.5612	0.051*	
C14	0.8577 (5)	0.3269 (2)	0.54661 (10)	0.0445 (7)	
H14A	0.9419	0.2920	0.5252	0.053*	
C17	1.3569 (6)	0.5691 (2)	0.51091 (12)	0.0553 (8)	
C8	0.4569 (4)	0.2927 (2)	0.70916 (10)	0.0446 (8)	
H8A	0.5896	0.3074	0.6958	0.053*	
H8B	0.4496	0.2250	0.7134	0.053*	
C9	0.3716 (5)	0.2892 (2)	0.61904 (10)	0.0452 (8)	
H9A	0.2633	0.3024	0.5950	0.054*	
H9B	0.3912	0.2216	0.6197	0.054*	
C2	−0.0731 (6)	0.2714 (2)	0.84573 (12)	0.0484 (8)	
C10	0.5610 (5)	0.3349 (2)	0.59923 (10)	0.0400 (7)	
C7	0.1125 (4)	0.2830 (2)	0.68263 (11)	0.0452 (7)	
H7A	0.1228	0.2150	0.6842	0.054*	
H7B	0.0237	0.2982	0.6541	0.054*	
C16	1.0824 (5)	0.4642 (2)	0.53409 (12)	0.0456 (8)	
C11	0.6024 (5)	0.4287 (2)	0.60916 (12)	0.0519 (9)	
H11A	0.5162	0.4639	0.6299	0.062*	
C13	0.9016 (4)	0.4206 (2)	0.55675 (11)	0.0420 (8)	
C12	0.7718 (6)	0.4699 (2)	0.58827 (12)	0.0543 (10)	
H12A	0.7992	0.5326	0.5957	0.065*	
O1	0.2794 (4)	0.08728 (18)	0.80003 (10)	0.0844 (9)	
C20	1.6675 (7)	0.5202 (3)	0.44691 (14)	0.0793 (12)	
H20A	1.7743	0.5041	0.4254	0.095*	
C1	0.2304 (5)	0.1636 (3)	0.80624 (12)	0.0552 (9)	
C18	1.5090 (6)	0.6342 (3)	0.50075 (15)	0.0742 (11)	
H18A	1.5064	0.6939	0.5155	0.089*	
C19	1.6602 (7)	0.6080 (3)	0.46892 (16)	0.0804 (12)	
H19A	1.7623	0.6508	0.4617	0.096*	
C21	1.5182 (6)	0.4561 (3)	0.45645 (14)	0.0680 (10)	
H21A	1.5217	0.3969	0.4410	0.082*	
O6	−0.2147 (4)	0.52106 (18)	0.81418 (11)	0.0758 (8)	
O5	0.2323 (5)	0.56723 (19)	0.68549 (12)	0.1013 (11)	
C6	0.2071 (5)	0.5196 (2)	0.72024 (16)	0.0636 (10)	
O4	0.3651 (4)	0.3247 (2)	0.91230 (9)	0.0943 (9)	
O3	0.3659 (5)	0.5584 (2)	0.85459 (13)	0.1067 (11)	
C5	0.2917 (6)	0.5140 (3)	0.82303 (16)	0.0676 (11)	
C4	−0.0683 (6)	0.4912 (2)	0.79849 (14)	0.0553 (9)	
C3	0.2877 (5)	0.3088 (3)	0.87403 (13)	0.0626 (10)	

### Atomic displacement parameters (Å^2^)

**Table:**

	*U*^11^	*U*^22^	*U*^33^	*U*^12^	*U*^13^	*U*^23^
Fe1	0.0384 (3)	0.0517 (3)	0.0384 (2)	−0.0004 (2)	−0.0018 (2)	0.0047 (2)
Fe2	0.0388 (2)	0.0445 (2)	0.0505 (3)	−0.0012 (2)	0.0048 (2)	−0.0011 (2)
S1	0.0310 (4)	0.0528 (4)	0.0425 (4)	−0.0009 (4)	−0.0030 (4)	0.0025 (4)
S2	0.0326 (4)	0.0577 (5)	0.0396 (4)	−0.0015 (3)	−0.0032 (4)	−0.0018 (4)
S3	0.0765 (7)	0.0494 (5)	0.0712 (6)	−0.0132 (5)	0.0074 (6)	−0.0109 (4)
N2	0.0531 (18)	0.0530 (16)	0.0450 (15)	−0.0094 (15)	0.0074 (14)	−0.0076 (13)
C22	0.049 (2)	0.064 (2)	0.0410 (18)	−0.0103 (19)	−0.0034 (18)	0.0065 (16)
N1	0.0319 (14)	0.0501 (14)	0.0316 (12)	0.0013 (12)	−0.0027 (11)	0.0013 (10)
O2	0.0529 (16)	0.0789 (17)	0.0634 (16)	−0.0022 (13)	0.0112 (14)	0.0070 (13)
C15	0.050 (2)	0.0389 (15)	0.0378 (16)	0.0008 (17)	−0.0024 (16)	−0.0075 (14)
C14	0.051 (2)	0.0440 (17)	0.0386 (16)	0.0019 (17)	0.0071 (17)	−0.0063 (14)
C17	0.062 (2)	0.057 (2)	0.0467 (19)	−0.017 (2)	−0.0046 (19)	0.0089 (16)
C8	0.0343 (17)	0.0570 (19)	0.0424 (18)	0.0067 (15)	0.0021 (14)	−0.0005 (15)
C9	0.044 (2)	0.0549 (18)	0.0363 (17)	0.0024 (18)	−0.0042 (15)	−0.0033 (14)
C2	0.055 (2)	0.0471 (19)	0.0430 (19)	−0.0033 (18)	−0.0045 (17)	0.0058 (15)
C10	0.0458 (19)	0.0441 (18)	0.0301 (15)	0.0010 (15)	−0.0034 (14)	−0.0016 (14)
C7	0.0414 (19)	0.0546 (18)	0.0397 (17)	−0.0037 (16)	−0.0051 (15)	−0.0016 (15)
C16	0.053 (2)	0.0458 (19)	0.0375 (18)	−0.0033 (16)	−0.0008 (16)	−0.0018 (14)
C11	0.053 (2)	0.053 (2)	0.049 (2)	−0.0013 (17)	0.0145 (17)	−0.0113 (16)
C13	0.048 (2)	0.0478 (18)	0.0301 (16)	−0.0038 (15)	0.0021 (14)	−0.0030 (14)
C12	0.072 (3)	0.0394 (18)	0.052 (2)	−0.0058 (17)	0.0101 (18)	−0.0087 (15)
O1	0.103 (2)	0.0630 (17)	0.087 (2)	0.0162 (16)	0.0040 (16)	0.0124 (14)
C20	0.057 (3)	0.121 (4)	0.059 (2)	−0.016 (3)	0.007 (2)	0.014 (2)
C1	0.051 (2)	0.069 (2)	0.0452 (19)	−0.0027 (19)	0.0031 (16)	0.0118 (18)
C18	0.073 (3)	0.069 (2)	0.082 (3)	−0.022 (2)	−0.010 (3)	0.013 (2)
C19	0.067 (3)	0.096 (3)	0.078 (3)	−0.033 (3)	−0.008 (3)	0.030 (2)
C21	0.057 (2)	0.089 (3)	0.058 (2)	−0.016 (2)	0.011 (2)	−0.008 (2)
O6	0.0529 (17)	0.0811 (19)	0.093 (2)	0.0149 (14)	0.0220 (15)	−0.0113 (15)
O5	0.121 (3)	0.0787 (19)	0.105 (2)	0.0319 (18)	0.0445 (19)	0.0460 (18)
C6	0.056 (2)	0.055 (2)	0.080 (3)	0.0155 (18)	0.013 (2)	0.005 (2)
O4	0.0653 (18)	0.170 (3)	0.0481 (15)	0.004 (2)	−0.0124 (14)	−0.0157 (17)
O3	0.079 (2)	0.118 (2)	0.123 (2)	−0.027 (2)	0.008 (2)	−0.067 (2)
C5	0.053 (3)	0.069 (2)	0.081 (3)	−0.007 (2)	0.017 (2)	−0.021 (2)
C4	0.061 (2)	0.0457 (19)	0.060 (2)	−0.0039 (18)	0.0030 (19)	0.0018 (17)
C3	0.048 (2)	0.093 (3)	0.047 (2)	0.004 (2)	0.0012 (18)	0.000 (2)

### Geometric parameters (Å, º)

**Table:**

Fe1—C1	1.778 (4)	C14—H14A	0.9300
Fe1—C3	1.787 (4)	C17—C18	1.395 (5)
Fe1—C2	1.798 (4)	C8—H8A	0.9700
Fe1—S1	2.2485 (11)	C8—H8B	0.9700
Fe1—S2	2.2487 (11)	C9—C10	1.506 (4)
Fe1—Fe2	2.5013 (12)	C9—H9A	0.9700
Fe2—C5	1.775 (4)	C9—H9B	0.9700
Fe2—C4	1.787 (4)	C10—C11	1.386 (4)
Fe2—C6	1.794 (4)	C7—H7A	0.9700
Fe2—S1	2.2465 (11)	C7—H7B	0.9700
Fe2—S2	2.2534 (11)	C16—C13	1.474 (4)
S1—C7	1.815 (3)	C11—C12	1.380 (4)
S2—C8	1.823 (3)	C11—H11A	0.9300
S3—C17	1.722 (4)	C13—C12	1.378 (4)
S3—C16	1.747 (3)	C12—H12A	0.9300
N2—C16	1.285 (4)	O1—C1	1.143 (4)
N2—C22	1.393 (4)	C20—C21	1.370 (5)
C22—C21	1.379 (5)	C20—C19	1.372 (5)
C22—C17	1.385 (4)	C20—H20A	0.9300
N1—C8	1.445 (3)	C18—C19	1.352 (6)
N1—C7	1.448 (4)	C18—H18A	0.9300
N1—C9	1.472 (3)	C19—H19A	0.9300
O2—C2	1.139 (4)	C21—H21A	0.9300
C15—C14	1.375 (4)	O6—C4	1.137 (4)
C15—C10	1.378 (4)	O5—C6	1.137 (4)
C15—H15A	0.9300	O4—C3	1.138 (4)
C14—C13	1.388 (4)	O3—C5	1.143 (4)
			
C1—Fe1—C3	100.12 (16)	C18—C17—S3	129.6 (3)
C1—Fe1—C2	99.64 (15)	N1—C8—S2	115.84 (19)
C3—Fe1—C2	91.32 (15)	N1—C8—H8A	108.3
C1—Fe1—S1	105.63 (11)	S2—C8—H8A	108.3
C3—Fe1—S1	154.05 (13)	N1—C8—H8B	108.3
C2—Fe1—S1	87.60 (11)	S2—C8—H8B	108.3
C1—Fe1—S2	98.12 (11)	H8A—C8—H8B	107.4
C3—Fe1—S2	88.79 (12)	N1—C9—C10	114.4 (2)
C2—Fe1—S2	161.94 (11)	N1—C9—H9A	108.7
S1—Fe1—S2	84.46 (4)	C10—C9—H9A	108.7
C1—Fe1—Fe2	147.16 (10)	N1—C9—H9B	108.7
C3—Fe1—Fe2	99.56 (12)	C10—C9—H9B	108.7
C2—Fe1—Fe2	105.92 (11)	H9A—C9—H9B	107.6
S1—Fe1—Fe2	56.15 (3)	O2—C2—Fe1	178.9 (3)
S2—Fe1—Fe2	56.34 (3)	C15—C10—C11	118.6 (3)
C5—Fe2—C4	89.06 (17)	C15—C10—C9	120.2 (3)
C5—Fe2—C6	99.18 (18)	C11—C10—C9	121.2 (3)
C4—Fe2—C6	100.78 (15)	N1—C7—S1	116.7 (2)
C5—Fe2—S1	157.95 (13)	N1—C7—H7A	108.1
C4—Fe2—S1	88.16 (11)	S1—C7—H7A	108.1
C6—Fe2—S1	102.83 (13)	N1—C7—H7B	108.1
C5—Fe2—S2	89.58 (13)	S1—C7—H7B	108.1
C4—Fe2—S2	156.54 (12)	H7A—C7—H7B	107.3
C6—Fe2—S2	102.55 (11)	N2—C16—C13	124.1 (3)
S1—Fe2—S2	84.40 (5)	N2—C16—S3	115.5 (2)
C5—Fe2—Fe1	103.08 (14)	C13—C16—S3	120.4 (2)
C4—Fe2—Fe1	101.48 (11)	C12—C11—C10	119.8 (3)
C6—Fe2—Fe1	148.57 (11)	C12—C11—H11A	120.1
S1—Fe2—Fe1	56.23 (3)	C10—C11—H11A	120.1
S2—Fe2—Fe1	56.16 (3)	C12—C13—C14	117.9 (3)
C7—S1—Fe2	110.81 (10)	C12—C13—C16	122.2 (3)
C7—S1—Fe1	113.84 (10)	C14—C13—C16	120.0 (3)
Fe2—S1—Fe1	67.62 (3)	C13—C12—C11	121.9 (3)
C8—S2—Fe1	109.38 (11)	C13—C12—H12A	119.1
C8—S2—Fe2	111.55 (10)	C11—C12—H12A	119.1
Fe1—S2—Fe2	67.50 (4)	C21—C20—C19	120.3 (4)
C17—S3—C16	89.05 (16)	C21—C20—H20A	119.9
C16—N2—C22	110.9 (3)	C19—C20—H20A	119.9
C21—C22—C17	119.6 (3)	O1—C1—Fe1	176.9 (3)
C21—C22—N2	125.6 (3)	C19—C18—C17	118.2 (4)
C17—C22—N2	114.7 (3)	C19—C18—H18A	120.9
C8—N1—C7	111.8 (2)	C17—C18—H18A	120.9
C8—N1—C9	110.6 (2)	C18—C19—C20	121.9 (4)
C7—N1—C9	109.4 (2)	C18—C19—H19A	119.0
C14—C15—C10	121.3 (3)	C20—C19—H19A	119.0
C14—C15—H15A	119.3	C20—C21—C22	119.4 (4)
C10—C15—H15A	119.3	C20—C21—H21A	120.3
C15—C14—C13	120.5 (3)	C22—C21—H21A	120.3
C15—C14—H14A	119.7	O5—C6—Fe2	179.3 (3)
C13—C14—H14A	119.7	O3—C5—Fe2	177.4 (4)
C22—C17—C18	120.6 (4)	O6—C4—Fe2	178.2 (3)
C22—C17—S3	109.8 (3)	O4—C3—Fe1	179.8 (4)
			
C1—Fe1—Fe2—C5	−124.5 (2)	C6—Fe2—S2—C8	−51.71 (17)
C3—Fe1—Fe2—C5	1.57 (17)	S1—Fe2—S2—C8	50.22 (11)
C2—Fe1—Fe2—C5	95.72 (16)	Fe1—Fe2—S2—C8	102.86 (12)
S1—Fe1—Fe2—C5	171.62 (12)	C5—Fe2—S2—Fe1	106.11 (13)
S2—Fe1—Fe2—C5	−80.50 (13)	C4—Fe2—S2—Fe1	19.5 (3)
C1—Fe1—Fe2—C4	143.7 (2)	C6—Fe2—S2—Fe1	−154.58 (13)
C3—Fe1—Fe2—C4	−90.15 (16)	S1—Fe2—S2—Fe1	−52.64 (4)
C2—Fe1—Fe2—C4	4.00 (16)	C16—N2—C22—C21	−180.0 (3)
S1—Fe1—Fe2—C4	79.90 (12)	C16—N2—C22—C17	0.1 (4)
S2—Fe1—Fe2—C4	−172.22 (12)	C10—C15—C14—C13	1.0 (4)
C1—Fe1—Fe2—C6	9.4 (3)	C21—C22—C17—C18	−1.0 (5)
C3—Fe1—Fe2—C6	135.5 (3)	N2—C22—C17—C18	178.9 (3)
C2—Fe1—Fe2—C6	−130.3 (3)	C21—C22—C17—S3	179.7 (3)
S1—Fe1—Fe2—C6	−54.4 (2)	N2—C22—C17—S3	−0.4 (4)
S2—Fe1—Fe2—C6	53.5 (2)	C16—S3—C17—C22	0.4 (3)
C1—Fe1—Fe2—S1	63.8 (2)	C16—S3—C17—C18	−178.8 (3)
C3—Fe1—Fe2—S1	−170.05 (11)	C7—N1—C8—S2	−70.5 (3)
C2—Fe1—Fe2—S1	−75.90 (11)	C9—N1—C8—S2	167.3 (2)
S2—Fe1—Fe2—S1	107.88 (5)	Fe1—S2—C8—N1	73.4 (2)
C1—Fe1—Fe2—S2	−44.0 (2)	Fe2—S2—C8—N1	0.7 (3)
C3—Fe1—Fe2—S2	82.07 (11)	C8—N1—C9—C10	−64.8 (3)
C2—Fe1—Fe2—S2	176.22 (11)	C7—N1—C9—C10	171.6 (2)
S1—Fe1—Fe2—S2	−107.88 (5)	C14—C15—C10—C11	−0.3 (4)
C5—Fe2—S1—C7	−130.3 (3)	C14—C15—C10—C9	175.7 (3)
C4—Fe2—S1—C7	146.74 (16)	N1—C9—C10—C15	146.5 (3)
C6—Fe2—S1—C7	46.09 (15)	N1—C9—C10—C11	−37.6 (4)
S2—Fe2—S1—C7	−55.54 (11)	C8—N1—C7—S1	63.7 (3)
Fe1—Fe2—S1—C7	−108.12 (11)	C9—N1—C7—S1	−173.4 (2)
C5—Fe2—S1—Fe1	−22.2 (3)	Fe2—S1—C7—N1	11.1 (2)
C4—Fe2—S1—Fe1	−105.14 (12)	Fe1—S1—C7—N1	−62.8 (2)
C6—Fe2—S1—Fe1	154.22 (11)	C22—N2—C16—C13	−178.7 (3)
S2—Fe2—S1—Fe1	52.59 (3)	C22—N2—C16—S3	0.3 (3)
C1—Fe1—S1—C7	−45.86 (16)	C17—S3—C16—N2	−0.4 (3)
C3—Fe1—S1—C7	126.7 (3)	C17—S3—C16—C13	178.6 (3)
C2—Fe1—S1—C7	−145.20 (15)	C15—C10—C11—C12	−0.7 (4)
S2—Fe1—S1—C7	51.04 (12)	C9—C10—C11—C12	−176.7 (3)
Fe2—Fe1—S1—C7	103.78 (12)	C15—C14—C13—C12	−0.6 (4)
C1—Fe1—S1—Fe2	−149.64 (11)	C15—C14—C13—C16	179.3 (3)
C3—Fe1—S1—Fe2	22.9 (3)	N2—C16—C13—C12	−177.5 (3)
C2—Fe1—S1—Fe2	111.02 (11)	S3—C16—C13—C12	3.6 (4)
S2—Fe1—S1—Fe2	−52.74 (3)	N2—C16—C13—C14	2.5 (5)
C1—Fe1—S2—C8	51.61 (15)	S3—C16—C13—C14	−176.3 (2)
C3—Fe1—S2—C8	151.65 (15)	C14—C13—C12—C11	−0.4 (5)
C2—Fe1—S2—C8	−117.8 (3)	C16—C13—C12—C11	179.7 (3)
S1—Fe1—S2—C8	−53.44 (11)	C10—C11—C12—C13	1.1 (5)
Fe2—Fe1—S2—C8	−106.01 (11)	C22—C17—C18—C19	0.4 (5)
C1—Fe1—S2—Fe2	157.61 (11)	S3—C17—C18—C19	179.6 (3)
C3—Fe1—S2—Fe2	−102.34 (12)	C17—C18—C19—C20	−0.2 (6)
C2—Fe1—S2—Fe2	−11.8 (3)	C21—C20—C19—C18	0.6 (6)
S1—Fe1—S2—Fe2	52.57 (3)	C19—C20—C21—C22	−1.2 (6)
C5—Fe2—S2—C8	−151.02 (17)	C17—C22—C21—C20	1.4 (5)
C4—Fe2—S2—C8	122.3 (3)	N2—C22—C21—C20	−178.6 (3)
